# Reduction in time to viral suppression among persons living with HIV in Jamaica between 2017–2019

**DOI:** 10.1371/journal.pgph.0003107

**Published:** 2024-04-25

**Authors:** Anya Cushnie, Ralf Reintjes, Miia Artama, J. Peter Figueroa

**Affiliations:** 1 Unit of Health Sciences, Faculty of Social Sciences, Tampere University, Tampere, Finland; 2 Department of Health Sciences, Hamburg University of Applied Sciences, Hamburg, Germany; 3 Department of Community Health and Psychiatry, University of the West Indies, Mona, Jamaica; Universidad de Chile, CHILE

## Abstract

**Introduction:**

HIV viral suppression is important for effective treatment and for reducing new infections. In 2019, only 66% of persons on antiretroviral treatment (ART) in Jamaica were virally suppressed. We aim to compare time to viral suppression by ART initiation year and type of treatment site to understand the implications for programming.

**Methods:**

We assessed time to viral suppression among 4560 persons who received viral load testing either pre or post ART initiation from 2017–2019. We used descriptive statistics and Kaplan-Meier estimates to compare survival curves by ART year (2017, 2018, 2019), sex and type of treatment site (public and non-governmental organizations). Persons were censored if suppression was not achieved. Mixed effects Cox regression was used to determine the effect of covariates on the likelihood of viral suppression. We report hazard ratios and 95% confidence intervals.

**Results:**

Pre-ART viral load testing decreased from 36% in 2017 to 30% in 2019. For post-ART viral load tests, approximately 78% (n = 1589) of persons achieved suppression, 51% (n = 809) were female and 86% (n = 1341) used a public treatment site. The median time to suppression decreased by 3 months from 2017 to 2019. The likelihood of suppression was almost 2 times greater in 2018 (HR = 1.56, CI = 1.39–1.75) and 3 times greater in 2019 (HR = 3.17, CI = 2.76–3.64) compared to 2017. NGO treatment sites were also significantly associated with the likelihood of viral suppression compared to public sites.

**Conclusion:**

Pre-ART viral load testing and the time to viral suppression decreased over three years. Initiating ART after 2017 and early use of NGO treatment sites were found to significantly increase the likelihood of achieving suppression. This demonstrates improvements in the national HIV response but there is need to increase the number of persons on ART and achieving viral suppression.

## Introduction

Jamaica has adopted the UNAIDS 90-90-90 targets to achieve 90% HIV diagnosis, 90% ART retention and 90% viral suppression by 2020 (now 95-95-95 by 2030) [1]. To support target achievement, the national HIV treatment guidelines were revised in 2017 to initiate HIV treatment immediately in all diagnosed patients regardless of clinical staging [2], as recommended by the 2015 WHO guidelines also known as the Treat All or Test and Start Strategy [3]. By 2019, 66% of HIV diagnosed persons on antiretroviral treatment (ART) were virally suppressed, leaving a gap of 34% who were unsuppressed and Jamaica did not achieve the global target of 90% at the end of 2020 [4].

The goal of HIV treatment is to achieve viral suppression and thus reduce mortality and morbidity associated with HIV infection. The level of viral suppression in a population determines the risk of viral transmission and new HIV cases [5]. This was demonstrated through several clinical studies [6] which led to the Undetectable = Untransmittable campaign (U = U) [7]. U = U means people with HIV who achieve and maintain an undetectable viral load by taking ART daily as prescribed are unlikely to sexually transmit the virus to others [8] Assessing the time to achieve suppression and identifying factors that may contribute to persons not achieving viral suppression can be used to improve their treatment outcomes.

Jamaica’s HIV epidemic is both general and concentrated among men who have sex with men (MSM) [9, 10]. Persons living with HIV(PLHIV) and MSM are at risk of being stigmatized in the public system [11], which presents a barrier to accessing HIV services. Broadening service provision to providers beyond the public system improves access for high-risk groups. Globally and locally, non-governmental organizations (NGOs), including community-based organizations, have a long record of health care provision and have been instrumental in providing robust, non-discriminatory HIV services to key populations [12, 13]. Currently, Jamaica delivers HIV treatment and care services through a decentralized network of 90 treatment sites (47 public government sites, 6 independent sites and 37 private physicians) [14]. Beyond the need to ensure services are provided to high risk populations, research has shown that expanding service providers improves overall patient retention [15, 16].

This is the third paper in a series of manuscripts that aim to assess HIV treatment outcomes for Jamaica based on various factors. In previous papers we found early HIV diagnosis and same day ART initiation were significantly associated with viral suppression but age showed no association. [15, 16]. This study assesses time to viral suppression over three years after the implementation of Treat All based on ART initiation year and the type of treatment site (public and NGO), with the objective of investigating temporal trends in HIV treatment outcomes.

## Methods

The study used retrospective anonymized secondary data, collected from the national treatment services information system (TSIS2). The initial sample consisted of 4560 persons, age 15 years or older, who initiated HIV treatment at a total of forty-three (43) public and non-governmental treatment sites between 2017–2019, for an overall study time of 36 months.

### Data analysis

We provide descriptive statistics for the initial sample based on when persons received viral load testing (pre and post ART vl test). We conducted a Survival analysis to compare median time to viral suppression (<1000 copies/mL), by ART initiation year (2017, 2018, 2019), sex and type of treatment site (public versus NGOs). The survival model included 2049 PLHIV with a recorded first viral load test post ART initiation, at twenty-nine public sites and nine non-governmental sites (three civil society organizations, and six semi-private sites that receive both public and private funding), for a total of thirty-eight (38) treatment sites. The sample included only the first recorded viral load test for each patient. Censure occurred if persons did not achieve viral suppression within the study period. A mixed effects Cox regression model was used to identify predictors of suppression incorporating random effects to account for treatment site variability [17]. We did not institute a variable selection strategy due to the limited number of covariates included. We report adjusted hazard ratio with 95% CI. All analysis was performed in R Programme version 3.6.3 [18] and regression analysis was performed using the *Finalfit* package [19].

### Variables of interest

1. Time to ART initiation was based on the difference (in days) between the first clinic date and the ART initiation date.
2. To explore survival time, the time to the first viral load test was based on the difference (in months) between the ART initiation date and the first recorded viral load test date after ART initiation (post-ART).
3. Event: Viral suppression (<1000 copies/mL within the study period).
4. Censure: Viral suppression not achieved during the study period.

### Ethical approval

Ethical approval was given by the Ministry of Health and Wellness (MoHW) (Study No: 2017/20). The dataset was extracted by the MoHW and fully anonymized before sharing with the study investigator, as a result patient consent was waived.

## Results

The initial sample (n = 4560) consisted mostly of females (n = 2560, 56%), ages 20–39 years (n = 2437, 54%) (Table 1). Most persons were diagnosed at an early HIV stage (CD4≥350 cells/mm^3^) (n = 2759, 65%), with a median ART initiation time of 14 days and just over half (n = 2372, 52%) had achieved viral suppression at the first recorded viral load test which was done < 1 month after ART initiation.

**Table 1:** Characteristics of PLHIV in Jamaica who initiated ARV treatment between 2017–2019 with pre and post ART viral load tests (N = 4560).

Variables		Pre-ART viral load tests (N = 2511)		Post-ART viral load test (N-2049)		N = 4506	
	Levels	n	%	n	%	n	%
Sex	Female	1509	60	1051	51	2560	56
	Male	1002	40	998	49	2000	44
ART initiation year	2017	926	36	807	39	1733	38
	2018	849	34	766	37	1615	35
	2019	736	30	476	23	1212	27
Age group at ART initiation/years	15–19	117	5	113	6	230	5
	20–39	1374	55	1063	52	2437	54
	40+	1001	40	865	42	1866	41
	*Missing*	19		8		27	
HIV stage at diagnosis^a^	early diagnosis (CD4≥350 cells/mm^3^)	1657	70	1102	58	2759	65
	late diagnosis (CD4<350 cells/mm^3^)	709	30	784	42	1493	35
	*Missing*	145		163		308	
Median time of ART initiation/days (IQR)^b^		69(1384)		0(61)		14(535)	
Median time of first viral load test/months (IQR)^c^		1.3(21)		5.6(4.9)		0.2(7)	
Median first viral load result/copies/mL (IQR)		7489(47918)		20(450)		658 (24526)	
Viral load status at first viral load test^d^	Suppressed	783	32	1589	78	2372	52
	Unsuppressed	1728	69	460	22	2188	48
Type of Treatment Site	Public	2119	85	1756	86	3875	85
	NGO	392	15	293	14	685	15

^a.^ Based on baseline CD4 test results: Patients starting ART with CD4 cell count ≥350 cells/mm3 were characterized as achieving early diagnosis, while CD4 <350 cells/mm3 was defined as late diagnosis.

^b.^ Timing of ART initiation/days is the time from first clinic date to ART initiation.

^c.^ Timing of VL test/month is the time from the ART initiation date to first VL test date.

^d.^ Based on the first viral load test result after initiating treatment, followed by categorization at that time period (suppressed <1000 copies/mL versus unsuppressed ≥1000 copies/mL).

*Percentages shown are of column totals.

*IQR of the medians are reported as the difference between Q3 and Q1.

Many persons received pre-ART viral load testing (n = 2511, 55%) and most persons used a public government site (n = 3875, 85%) (Table 1). For those with pre-ART viral load testing, the demographic distribution was similar to the initial sample with a median ART initiation time of 69 days after the first clinic visit and a median viral load test time of 1 month before ART initiation. Most persons were virally unsuppressed at the first viral load test (n = 1728, 69%), despite most of them receiving an early HIV diagnosis (n = 1657, 70%).

For persons with post-ART viral load test (n = 2049, 45%), half (n = 809, 51%) were female who similarly mostly used public treatment sites (n = 86%, 1756) (Table 1). ART initiation occurred at the first clinic visit testing (0 days or same day initiation) and the median time for the first recorded viral load test was approximately 6 months after ART initiation with 78% (n = 1589) achieving suppression at the first test.

### Survival analysis for persons with post-ART viral load tests

**Fig 1:**
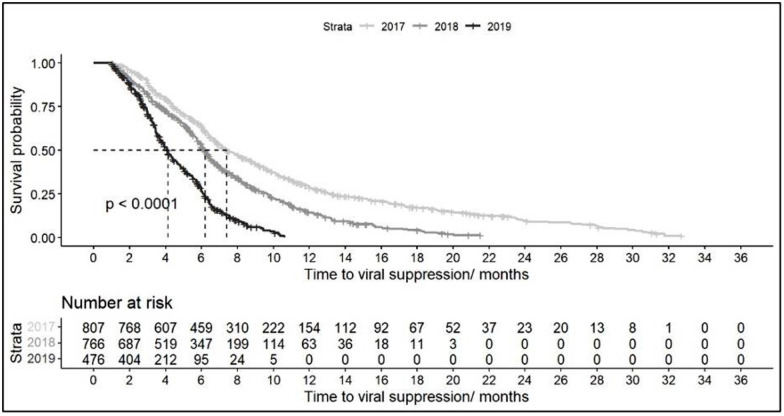
Kaplan-Meier curve for time to viral suppression stratified by year of ARV initiation.

**Fig 2:**
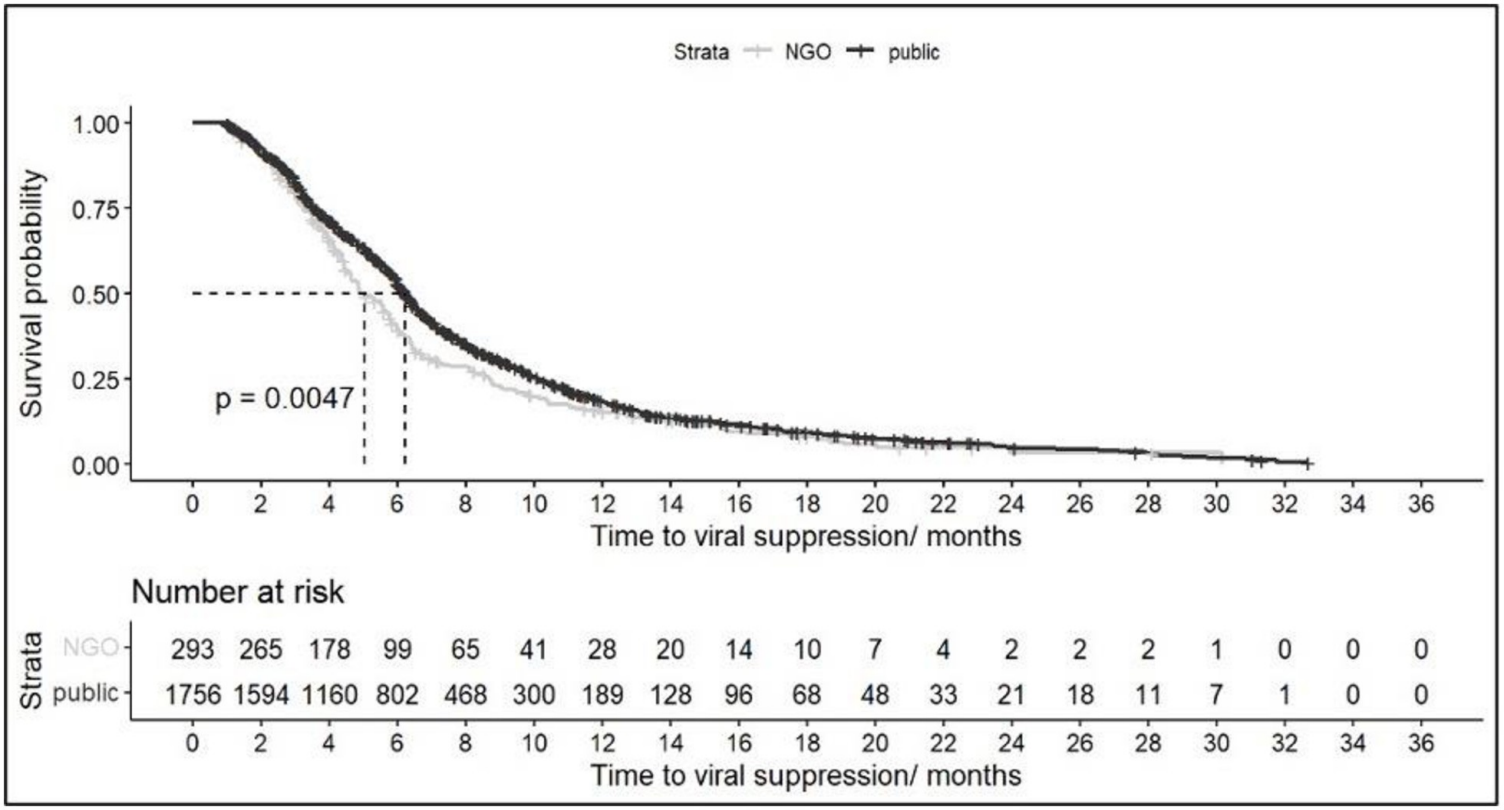
Kaplan-Meier curve for time to viral suppression stratified by type of treatment site (public versus NGOs).

We used a survival model to assess median time to viral suppression for persons with a vl test post ART initiation (n = 2049, 45%), and the median time to viral suppression decreased from 7.4 months (CI = 6.94–8.09) in 2017 to 4 months (CI = 3.85–4.37) in 2019 (Table 2). Patients using NGO treatment sites achieved suppression at a median time of 5 months (CI = 4.60–5.75) compared to 6.2 months (CI = 6.05–6.44) for those using public sites, however, after 12 months the time to suppression was similar between sites. There was no difference according to sex. This is further visualized in the Kaplan Meier Curves below (Figs 1 and 2). The median time to ARV initiation was 0 days and 460 persons were censored or did not achieve suppression during the study period.

**Table 2:** Median time to viral suppression among persons with post-ART viral load tests in Jamaica by ARV initiation year, sex and treatment site, 2017–2019 (N = 2049).

Variables	Levels	Patients n (%)	virally suppressed at the first viral load test (n) N = 1589	Median time to viral suppression after ART initiation/months (95% CI)
**ARV initiation year**	2017	807 (39)	600	7.40 (6.94–8.09)
	2018	766 (38)	606	6.21(5.98–6.44)
	2019	476 (23)	383	4.14 (3.85–4.37)
**Sex**	Female	1051 (51)	809	6.08 (5.88–6.38)
	Male	998 (49)	768	6.08 (5.98–6.38)
**Age at ARV initiation/years**	Mean (SD): 38 (13.5)			
**Type of treatment site**	public	1756(86)	1341	6.21(6.05–6.44)
	NGO	293(14)	236	5.03(4.60–5.75)

The Kaplan-Meier curves (Figs 1 and 2) display the time to achieve viral suppression after ART initiation stratified by year and type of treatment site. The curve shows overall the proportion of persons virally unsuppressed decreased over time. The horizontal dashed line at survival probability 50% is the estimated median time to viral suppression.

We assessed the probability of suppression at various times (S1 Table). At 3 months, 22% of persons (n = 354) achieved suppression at this time. After 6 months on treatment, this increased to 50% probability and 71% at 9 months. In 2019, the probability of suppression at 6 months was 75%, compared to 50% in 2018 and 40% in 2017. There was also a 60% probability of suppression for patients using NGO sites compared to 45% for public sites. Figures showing the cumulative incidence stratified by ART year and type of site are provided in the Supplement (S1 and S2 Figs).

Using a random effects cox regression model to account for heterogeneity by treatment site, ART initiation year and type of treatment site showed a significant association with viral suppression based on significance level of p<0.05 (Table 3). The likelihood of viral suppression was 1.5 times greater in 2018 (95%CI = 1.39–1.75) and 3 times greater in 2019 (95%CI = 2.76–3.64), compared to 2017. Persons using NGO sites had a greater likelihood of viral suppression than those using public sites. The heterogeneity between different site types, quantified by the variance of the random effect, was significant (p = 0.005). Sex was not significantly associated with the likelihood of viral suppression.

**Table 3:** Mixed effects cox regression showing predictors of viral suppression using hazard ratios among persons with post-ART viral load tests in Jamaica, 2017–2019.

Covariate	CHR (95%CI), p value	AHR (95% CI), p value
**Sex**		
female	ref	ref
male	0.94(0.86–1.04), p = 0.25	0.93(0.8401.03), p = 0.35
**ARV initiation year**		
2017	ref	ref
2018	1.57(1.40–1.76), p<0.001	1.56 (1.39–1.75), p<0.001
2019	3.17(2.76–3.64), p<0.001	3.17 (2.76–3.64), p<0.001
**Type of Treatment Site**		
Public government site	ref	ref
NGO site	1.22(1.06–1,40), p = 0.005	1.23(1.07–1.42), p = 0.004

## Discussion

We assessed all PLHIV who initiated treatment between 2017–2019 based on when viral load testing was received (pre and post ART viral load test). Most persons were diagnosed at an early HIV stage (CD4>350cells/mm3), started ART within 14 days of diagnosis, received a viral load test <1 month before initiating treatment and were virally suppressed at the first vl test. Most also used a public treatment site. Our results show pre-ART viral load testing has continued, mostly in the public sector, but decreased each year from 36% in 2017 to 30% in 2019. For persons with a pre-ART viral load test, although most had received an early HIV diagnosis, there was an overall delay in ART initiation of just over 2 months. The delay may be a result of physicians awaiting a baseline viral load test prior to starting patients on treatment or patients themselves delaying. One of the key factors for patients delaying treatment is not feeling sick [20] and since most of the sample was diagnosed at an early stage then they may not yet be symptomatic. The first recorded viral load test was conducted at a median time of 1 month prior to ART initiation and 69% were virally unsuppressed at this time, indicating a need for persons to be placed on treatment immediately once diagnosed. A higher viral load is associated with decreased likelihood of achieving suppression once on ART, longer times to suppression and infectiousness [21]. These results also mean some 30% of pre-ART viral load testers were virally suppressed. This could possibly be explained by patients silently transferring care from private to public care. At the time of data extraction, patient transfers were not recorded in TSIS2 nor was data from private sector sites included. Recognizing the significant gap in the data, in 2021 the national HIV program began increasing efforts to collect data from the network of private sector HIV providers [22]. This will also help to address possible misclassification of patients lost to follow up.

To investigate treatment effects, we assessed time to viral suppression for persons with post ART viral load tests using a survival model, stratified by year of ART initiation and the type of treatment site (public vs NGO). The median time to viral suppression improved from 7.4 months in 2017 to 4 months in 2019. Improving time to suppression is critical as this decreases the risk of viral transmission. This decrease may be attributed to implementation of the WHO Treat All Strategy, which was introduced in 2017 with the overall objective of increasing immediate ART initiation [2]. For post ART viral load tests, the median time to ARV initiation was 0 days or same day start. In a previous study, we showed same day ART initiation increased from 31% in 2017 to 51% in 2019 and was significantly associated with viral suppression [16]. After 2019, other strategies were implemented to increase viral suppression for persons on ART. These strategies have included multi-month dispensing of ART aimed at reducing the frequency of refills and increasing adherence [23] as well as transiting patients to tenofovir/lamivudine/dolutegravir (TLD) as a first line regimen which has been demonstrated to suppress viral load more quickly [24].

Examining the probability of suppression over time, we found persons mostly received a first viral load test at 3 months but only 21% were virally suppressed at this time. There was increased probability of achieving suppression at 6 and 9 months. While we do expect an increasing probability of suppression the longer persons are on treatment, this finding also indicates patients may be receiving a viral load test too soon at 3 months and adhering to the national guidelines of testing at 6 months would suffice, recognizing that repeated virologic monitoring of patients is challenging and Jamaica currently utilizes only one central laboratory to provide these services. In previous studies, we demonstrated that after 2017, there was an increase in median baseline CD4 count for PLHIV, indicating an increase in early HIV diagnoses (CD4 >350 cells/mm^3^) and most persons initiated ART on the same day diagnosed [15]. This may mean administering viral load testing at 6 months would be sufficient for patient management as persons are being diagnosed and treated faster, before they are symptomatic. However, treatment adherence should also be considered as this does have implications for treatment failure [20].

The decrease in time to suppression and increased probability over time may also be a result of improved retention on ART. In 2019, Jamaica achieved 52% retention on treatment among persons diagnosed with HIV [25]. Although this figure is difficult to qualify considering factors such as migration, which is high in Jamaica, it is notably much lower than the 90% (now 95%) global target [26], but an improvement from 42% in 2017 [25]. Improved retention may also account for persons having three times greater likelihood of achieving viral suppression in 2019 compared to 2017.

The study showed most persons used a public treatment site. Under 12 months on treatment, there was a difference of 1 month in the time to suppression between public and NGO sites. The median time to suppression was 5 months at NGO sites compared to 6 months at public sites. However, the time to suppression becomes comparable after 12 months on treatment. Based on the random site effect model, there was significant variability in time to suppression between sites. The NGO sites included in this study are community-based and public/private. The Global AIDS Strategy envisages that by 2025, 30% of HIV testing and treatment services will be delivered by community-led organizations [27]. Locally, HIV case notifications are highest among high-risk populations that are more likely to be stigmatized in the public system and therefore turn to the NGOs providers for an alternate means of non-discriminatory service delivery. The variability between NGO and public sites indicates NGO sites have been instrumental in improving HIV treatment outcomes and can be further leveraged. No difference was noted between sexes for time to suppression.

We recognize the strengths and limitations of the study. The analytical sample size is large and includes all persons initiated on treatment over a 3-year period. All persons had a first viral load test recorded within the study period, so no-one was excluded based on a missing test. Persons were censored if viral suppression was not achieved within the study time. Overall, 460 persons (22%) were censored and this decreased by 55% over 3 years. Censoring was random and non-informative [28, 29], which reduced bias. However, the national HIV treatment guidelines stipulate a first viral load test should be scheduled at 6 months after ART initiation [2]. This may explain why most persons using public treatment sites achieved suppression at this time specifically. Persons with pre-ART viral load testing were not included in the Survival model, because data was not provided on multiple viral load tests. Data was unavailable to allow analysis of follow up viral load tests, frequency of visits to treatment sites and ART retention. We also did not assess patients who may be lost to follow up or the effect of type of ART as 93% of the sample was on a tenofovir/lamivudine/efavirenz (TLE) regimen for the study period. Private sector provider data was also not included. The ART regimen was similar across all sites and did not change during the study period. We anticipated differences between public and NGO sites due to differences in operational factors (such as resources and location) and therefore accounted for clustering using a mixed effects cox regression model. Model concordance was reported at 60%.

## Conclusion

Pre-ART viral load testing has decreased but there is need to increase immediate treatment initiation. The decrease in time to viral suppression noted over 3 years demonstrates improvements in the national HIV response after changes were made in the national treatment guidelines to implement the Treat All Strategy. Initiating ART after 2017 and early use of NGO treatment sites were found to increase the likelihood of achieving suppression. The study shows there is programmatic value in monitoring temporal trends in time to viral load suppression. Jamaica needs to scale up the number of persons on ART and achieving viral suppression. Further studies should be conducted to assess the impact of viral suppression on HIV incidence and the private sector contribution to improving HIV treatment outcomes as part of Jamaica’s multisectoral response.

## Supporting information

S1 Table Probability of viral suppression over time, for 2017–2019. (DOCX)

S1 Fig Cumulative probability of viral suppression by ART year of initiation. (TIF)

S2 Fig Cumulative probability of viral suppression by treatment site. (TIF)
