# Supplementary material for: Reduction in time to viral suppression among persons living with HIV in Jamaica between 2017–2019
Source: PLOS Glob Public Health. 2024 Apr 25;4(4):e0003107. doi: 10.1371/journal.pgph.0003107 (PMC11045129; doi:10.1371/journal.pgph.0003107)
Supplement: S1 Table — (DOCX) [file pgph.0003107.s001.docx]

**S1 Table. Probability of viral suppression over time, for 2017-2019**

| **time/months** | **Number at risk** | **Number of events** | **Survival probability** | **std. error** | **lower 95% CI** | **upper 95% CI** |
| --- | --- | --- | --- | --- | --- | --- |
| 0 | 2049 | 0 | 1 | 0 | 1 | 1 |
| 3 | 1610 | 354 | 0.82 | 0.0085 | 0.8 | 0.84 |
| 6 | 901 | 583 | 0.51 | 0.011 | 0.48 | 0.53 |
| 9 | 425 | 356 | 0.29 | 0.011 | 0.27 | 0.31 |
| 12 | 217 | 157 | 0.18 | 0.009 | 0.15 | 0.19 |
| 18 | 78 | 92 | 0.09 | 0.008 | 0.08 | 0.10 |
| 24 | 23 | 31 | 0.05 | 0.007 | 0.03 | 0.06 |
| 30 | 8 | 11 | 0.02 | 0.006 | 0.01 | 0.04 |
