# Supplementary figures and images for: Reduction in time to viral suppression among persons living with HIV in Jamaica between 2017–2019
Source: PLOS Glob Public Health. 2024 Apr 25;4(4):e0003107. doi: 10.1371/journal.pgph.0003107 (PMC11045129; doi:10.1371/journal.pgph.0003107)

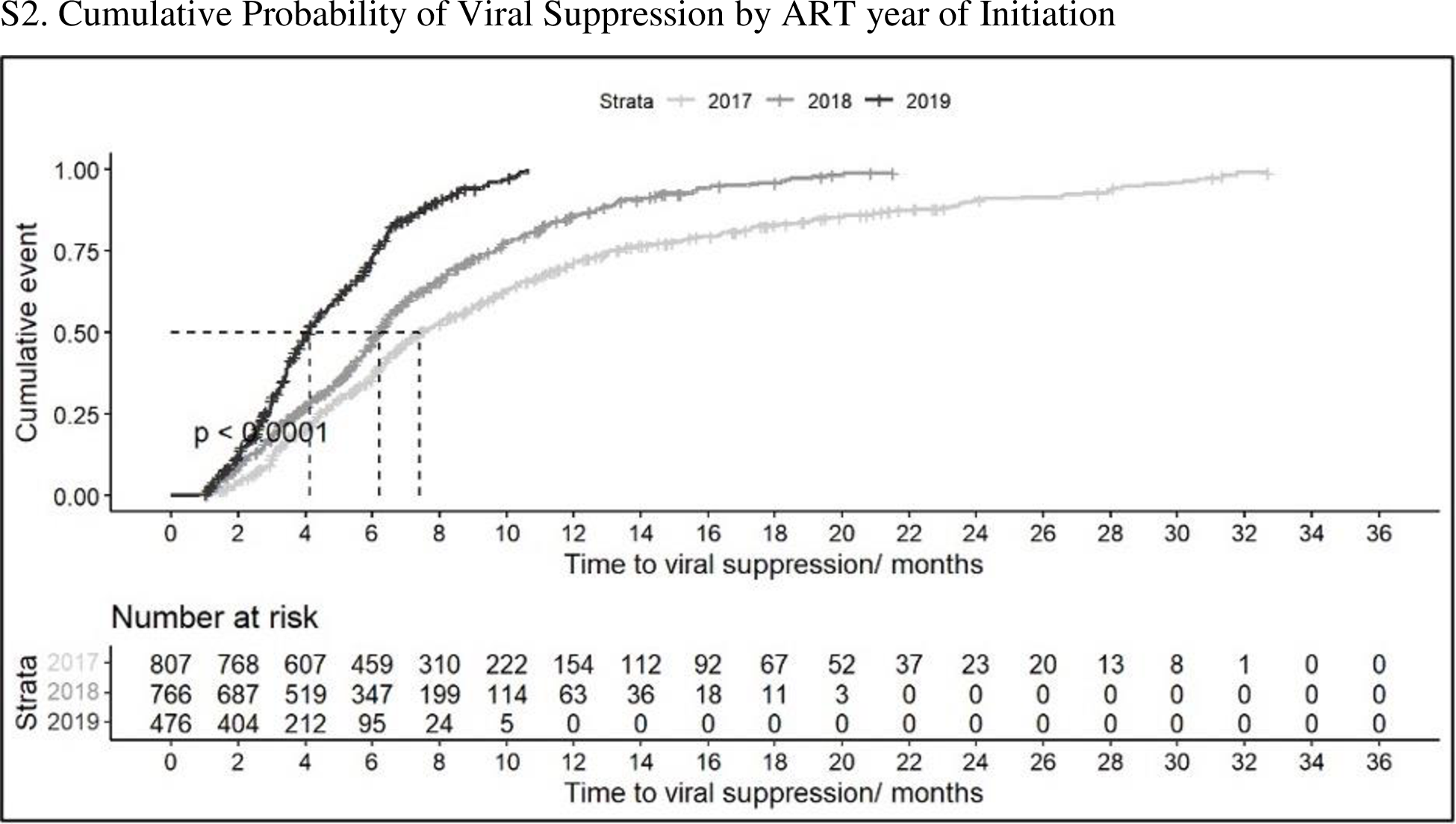

Supplement: S1 Fig — (TIF) [file pgph.0003107.s002.tif]

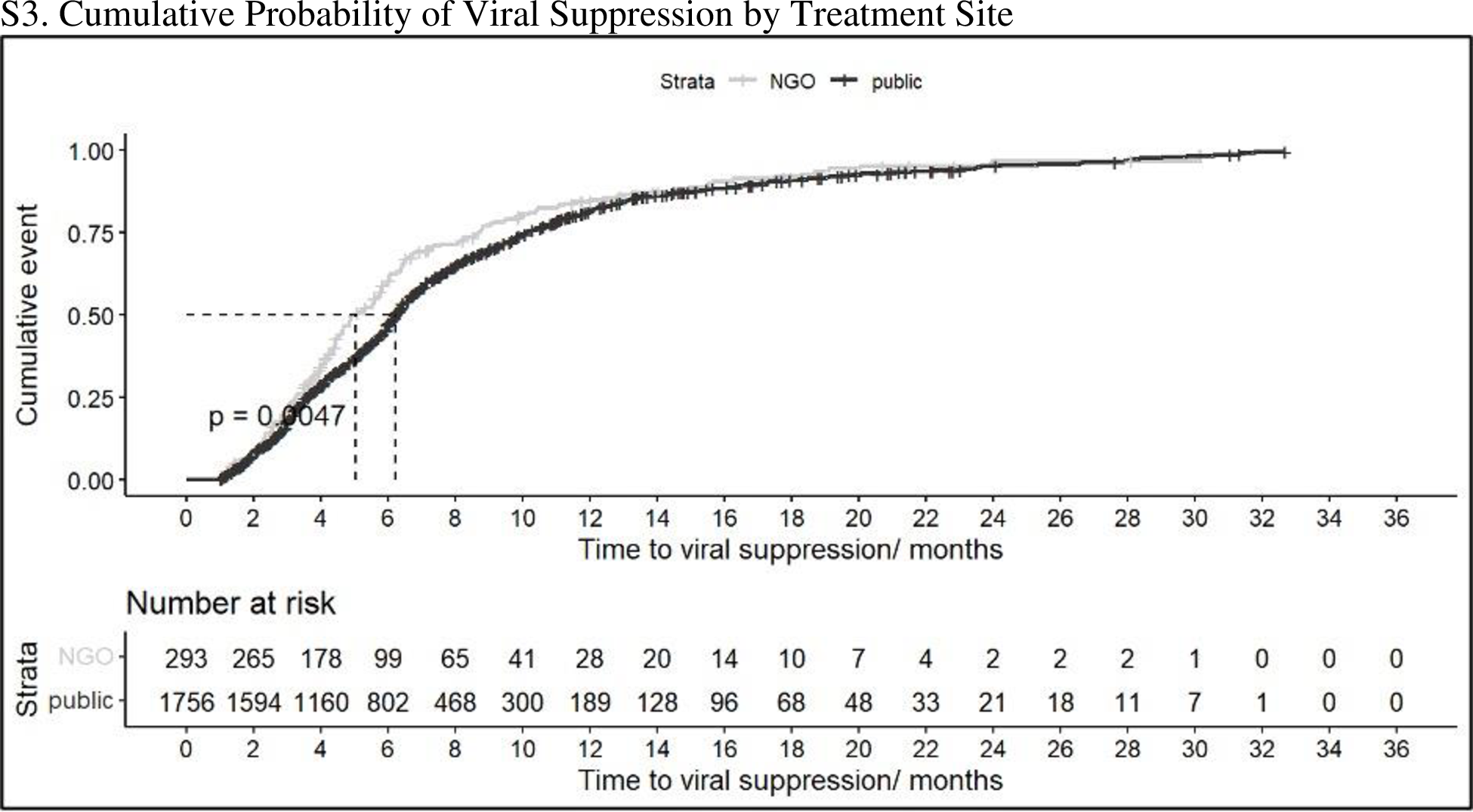

Supplement: S2 Fig — (TIF) [file pgph.0003107.s003.tif]
